# Latest advances in mechanisms of epileptic activity in Alzheimer’s disease and dementia with Lewy Bodies

**DOI:** 10.3389/fneur.2024.1277613

**Published:** 2024-02-08

**Authors:** Mariane Vicente, Kwaku Addo-Osafo, Keith Vossel

**Affiliations:** Mary S. Easton Center for Alzheimer’s Research and Care, Department of Neurology, David Geffen School of Medicine at University of California, Los Angeles, CA, United States

**Keywords:** Alzheimer’s disease, dementia with Lewy bodies, epilepsy, network hyperexcitability, epileptic activity

## Abstract

Alzheimer’s disease (AD) and dementia with Lewy bodies (DLB) stand as the prevailing sources of neurodegenerative dementia, impacting over 55 million individuals across the globe. Patients with AD and DLB exhibit a higher prevalence of epileptic activity compared to those with other forms of dementia. Seizures can accompany AD and DLB in early stages, and the associated epileptic activity can contribute to cognitive symptoms and exacerbate cognitive decline. Aberrant neuronal activity in AD and DLB may be caused by several mechanisms that are not yet understood. Hyperexcitability could be a biomarker for early detection of AD or DLB before the onset of dementia. In this review, we compare and contrast mechanisms of network hyperexcitability in AD and DLB. We examine the contributions of genetic risk factors, Ca^2+^ dysregulation, glutamate, AMPA and NMDA receptors, mTOR, pathological amyloid beta, tau and α-synuclein, altered microglial and astrocytic activity, and impaired inhibitory interneuron function. By gaining a deeper understanding of the molecular mechanisms that cause neuronal hyperexcitability, we might uncover therapeutic approaches to effectively ease symptoms and slow down the advancement of AD and DLB.

## Introduction

Hyperexcitability can be defined as an increased likelihood of firing at the level of the neuron from certain stimuli and/or due to decreased firing thresholds (1). This heightened excitability can clinically present itself as epilepsy. As per the official definition by the International League Against Epilepsy (2), “Epilepsy is characterized by repeated spontaneous bursts of neuronal hyperactivity and high synchronization in the brain.” Epilepsy has emerged as a significant global health issue, impacting approximately 70 million individuals worldwide (3–6). Hyperactivity occurs in neuronal populations or brain regions when the frequency of activity is above normal rates. Brain activity is normally regulated with precise timing and regional specificity, however, high synchronization or hypersynchrony denotes an increase in neuronal coordination and cellular firing (7, 8). While epilepsy can manifest in any stage of life, it is notably more common among individuals aged 65 years and older, reaching a prevalence of 5.7% in the Cardiovascular Health Study (9). Increasingly, there is a growing recognition that late-onset epilepsy, starting after age 55, is often not an isolated condition but is frequently linked to neurodegenerative diseases like Alzheimer’s disease (AD) and dementia with Lewy bodies (DLB) (10–12).

AD constitutes 60–70% of all dementia cases and is characterized by a gradual decline in memory and other cognitive functions. At present, there are more than 57 million people globally living with dementia, and this figure is predicted to double every two decades, reaching 74.7 million by 2030 (Alzheimer’s Disease International). The buildup of extracellular clusters of amyloid beta (Aβ) plaques and intracellular neurofibrillary tangles (NFTs) consisting of hyperphosphorylated tau protein in the cortical and limbic regions of the human brain signifies the disease’s pathological features (13–17). The accumulation of Aβ plaques and NFTs is connected with notable loss of neurons and synapses, along with neuroinflammation (18). In this context, there is a growing number of studies showing that patients with AD exhibit epilepsy, which may be a harbinger or indicator of the disease (11, 19–24). The prevalence of epilepsy in patients with AD is around 10 to 22% (21, 25, 26), while epileptiform activity, with varying characteristics, can be detected in patients with AD and with or without diagnosed epilepsy (23, 27–34). Seizures can begin in preclinical or clinical stages of AD (20, 23, 35, 36). The preponderance of seizures in AD lacks motor characteristics, rendering their diagnosis complex and potentially leading to an underreporting of seizures (23, 36, 37). Some studies suggest seizures can increase the production and deposition of Aβ and hyperphosphorylated tau in the brain and cause a decline in cognition in patients with AD (24, 38–41). Late-onset epilepsy increases risk of AD by around three-fold (12, 42). Notably, AD predisposes patients to develop epilepsy and late-onset epilepsy predisposes patients to develop AD highlighting the bidirectionality between diseases (11, 19).

DLB ranks as the second most frequent neurodegenerative dementia among individuals above the age of 65 (43–45). Clinical criteria encompass cognitive fluctuations, visual hallucinations, rapid eye movement sleep behavior disorder, and parkinsonism (45, 46). The neuropathology of DLB is marked by neuronal Lewy bodies and Lewy neurites, consisting of aggregates of α-synuclein that impact the brainstem along with extensive limbic and neocortical areas (47). This pathology also involves the loss of midbrain dopamine cells and cholinergic neurons in ventral forebrain nuclei, nucleus basalis of Meynert (48, 49). Furthermore, Aβ plaques and NFTs are present in a majority of DLB cases (50, 51). Analogous to AD, individuals with DLB also experience seizures (52). Marawar et al. (53) demonstrated a higher occurrence of seizures in DLB compared to the general population, with a rate of 3.8% in pathologically confirmed DLB across the United States. Meanwhile, Beagle et al. (52) identified a cumulative probability of 14.7% for DLB patients to develop seizures and a 5.1% prevalence of new-onset seizures in a population from the Memory Aging Center at the University of California, San Francisco, while other studies observed a 2–3% seizure prevalence rate in cohorts from Italy, United States, and Sweden (53–55).

**Figure 1:**
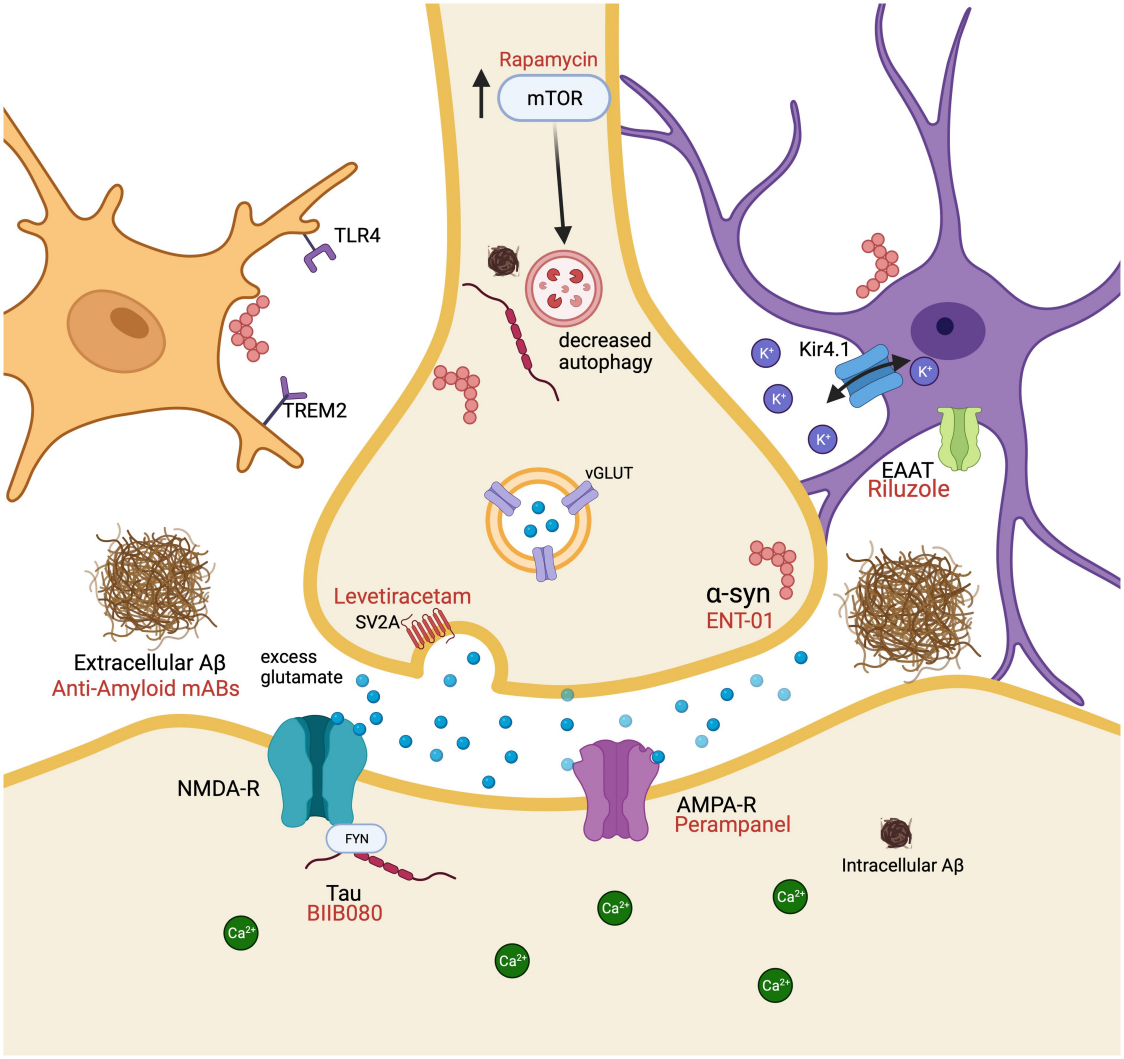
Molecular mechanisms resulting in cellular hyperexcitability associated with Alzheimer’s disease and dementia with Lewy bodies in a glutamatergic neuron surrounded by a microglial cell (peach) and an astrocyte (purple). Pharmacological interventions (red) are displayed by their receptor or protein of action. Aβ, amyloid beta; AMPA-R, α-amino-3-hydroxy-5-methyl-4-isoxazolepropionic acid; EAAT, excitatory amino acid transporters; mAbs, monoclonal antibodies; NMDA-R, N-methyl-D-aspartate receptor; TLR4, toll like receptor 4; TREM2, triggering receptor expressed on myeloid cells 2; mTOR, mechanistic target of rapamycin; SV2A, synaptic vesicle glycoprotein 2A; vGLUT, vesicular glutamate transporters. Created with BioRender.com.

In spite of the presence of antiseizure medications, roughly a third of individuals with epilepsy are unable to manage their seizures or develop resistance to the impact of these medications (56–59). This underscores the urgent need to create novel and inventive treatment approaches for epilepsy. Beyond that, therapeutic interventions targeting the molecular mechanisms of neuronal hyperexcitability have promise for treating disorders linked to increased excitability, such as AD and DLB. For example, Vossel et al. (60) showed that low doses of levetiracetam can improve spatial memory and executive function in AD patients with detectable epileptic activity. Levetiracetam also improved attention, oral fluency, and overall cognition in AD patients in a case–control study (61). Also, the clinical trial HOPE4MCI (NCT03486938) uses low dose levetiracetam which has been shown to decrease hippocampal hyperexcitability and attenuate cognitive decline by improving task related memory performance in amnestic mild cognitive impairment (62, 63). These studies show that levetiracetam can improve diverse cognitive functions in various stages of AD, reflecting multiple cortical regions that exhibit hyperexcitability in the disease. As a potential marker of neurodegeneration and pathology progression in AD and DLB, the early detection of cortical hyperexcitability and its mechanistic understanding is instrumental. Hyperexcitability may begin or be a result of neuropathology and may arise due to a number of different factors at varying time points in AD and DLB. Though hyperexcitability has been previously explored in the context of AD (1, 24, 31, 60, 64, 65), the role and mechanisms of hyperexcitability in DLB (66–69), as well as its similarities and differences with AD requires more research. In this review, we explore shared and distinct molecular mechanisms associated with hyperexcitability in AD and DLB, encompassing factors such as genetic risk factors, Ca^2^ and glutamate contributions, cholinergic pathways, AMPA and NMDA receptors, mTOR, pathological Aβ, tau and α-synuclein, genetic risk factors, altered microglial and astrocytic activity, and impaired inhibitory interneuron function (Figure 1).

### Genetic risk factors

#### APOE

The apolipoprotein E (APOE) ε4 allele is implicated in cerebrovascular, mental, and neurological disorders but stands as the primary genetic susceptibility factor for AD, and also increases the severity of neuropathology in DLB (70–75). In the context of hyperexcitability, APOE ε4 (APOE4) has not been associated with early-onset epilepsy, within 12 months of age, (76), but APOE4 has been linked to an increased risk of late-onset epilepsy, starting after age 60, and there exists an allele dose dependence on the incidence of late-onset epilepsy of 2.87, 4.13, and 7.05 per 1,000 person-years for 0, 1, and 2 APOE ε4 alleles, respectively (42, 77). These results persisted when participants with strokes were censored, suggesting that APOE4 confers epilepsy risk through mechanisms beyond its effects on cerebrovascular disease (42). A meta-analysis demonstrated that individuals carrying the APOE4 allele and experiencing temporal lobe epilepsy exhibit seizure onset nearly 4 years earlier than those without the allele (78). Another investigation revealed that individuals with temporal lobe epilepsy and APOE4 have an elevated risk of experiencing verbal learning deficits, particularly among those with a longer epilepsy duration (79). Similarly, mice expressing the human APOE4 allele develop a seizure phenotype that is either absent or less pronounced in mice expressing human APOE2 or APOE3 (80).

The exact mechanisms by which APOE4 promotes heightened neural excitability remain to be fully elucidated. APOE is involved in cholesterol metabolism and transportation, stabilization and solubilization of lipoproteins, and maintaining lipid homeostasis. Additionally, it plays a role in synaptic plasticity, signal transduction, and immunomodulation (81–83). *In vitro* studies utilizing human induced pluripotent stem cell-derived neurons expressing APOE4 demonstrate increased excitability compared to APOE3 isogenic controls. This heightened excitability might be attributed to an elevated expression of synaptic proteins like synaptophysin and PSD-95, the upregulation of genes involved in neuronal differentiation, and alterations in cholesterol metabolism (84). Due to the critical importance of APOE in shaping neuronal structure, establishing synapses, and regulating ion channels, changes in cholesterol and lipid concentrations can significantly impact neural excitability (85). For example, in rat hippocampal neurons, changed cholesterol levels differentially affect fast transient currents and delay rectifying currents modulating hyperexcitability (86). A clinical demonstration of importance is evident in Niemann-Pick type C (NPC) disease. In NPC, dysregulation of cholesterol transport and accumulation, can result in an AD-like phenotype, including cortical neurodegeneration, tau hyperphosphorylation, Aβ deposition, and hyperexcitability (87, 88). Vivas et al. (88) has shown that decreased transport of cholesterol from lysosomes disrupts ion channel activity and ultimately results in neuronal hyperexcitability. This mechanism is mediated by the reduction in phosphatidylinositol 4,5-bisphosphate in the plasma membrane resulting in a decrease in KCNQ2/3 current and increased excitability (88). Finally, microglia and astrocytes harboring APOE4 exhibit slowed uptake of extracellular Aβ (84). Consequently, elevated Aβ levels can also lead to increased neural activity. The plethora of physiological functions APOE is involved in results in numerous pathways by which APOE can contribute to hyperexcitability and targeted with therapeutics in AD and DLB (Table 1).

**Table 1:** Summary of molecular mechanisms cause hyperexcitability and intervention strategies associated with Alzheimer’s disease and dementia with Lewy Bodies.

Molecular mechanisms	Cause of hyperexcitability	Intervention strategies
Cholinergic pathways	Increased cholinergic tone before symptom onset	Cholinergic receptor antagonist during preclinical stages of AD or DLB
Excess glutamate	Excessive Ca^2+^ influx Overstimulation AMPA and NMDA receptors	Increase transporters (EAAT1, EAAT2, vGLUT1/vGLUT2) (e.g., riluzole) Antagonists of ionotropic and metabotropic glutamate receptors (e.g., perampanel) Antiseizure medications – SV2A mechanism (e.g., leviteracetam, brivaracetam)
Overactive mTOR	Reduced autophagy; buildup of epileptogenic disease proteins	Inhibition of mTOR (e.g., rapamycin)
Higher levels of α-synuclein	Overactivation of astrocytes and microglia	Inhibitors of aggregation (e.g., ENT-01) Inactivation of astrocytes and microglia (e.g., minocycline)
Tau protein	Enables seizures Can facilitate presynaptic glutamate release	Tau reduction (e.g., BIIB080)
Amyloid beta (Aβ)	Changes in voltage-dependent channels that maintain neuronal membrane potential Stimulation of voltage-gated calcium channels Formation of pores in the membrane thereby increasing Ca^+^ influx	Antibody-mediated clearance (e.g., anti-amyloid monoclonal antibodies) Inhibitors of aggregation Inhibitors of voltage-gated calcium channels
Over-stimulation of microglia and astrocytes	Increases glutamate release Decreases levels of the astrocytic glutamate transporter EAAT2 Endocytosis of neuronal ionotropic GABA_A_ receptors Activation of TLR4 receptors Increases extracellular K+ levels by astrocytes	Glial inhibition (e.g., minocycline) Increase glutamate transporters (e.g., ceftriaxone)
GABAergic neuron dysfunction	Mutations in genes encoding GABA receptor subunits Decreases voltage-gated sodium channels	Medications that increaese GABAergic tone (e.g., gabapentin and pregabalin)
Genetic risk factors: *APOE* ε4, *APP*, *PSEN1, PSEN2*, Trisomy 21, *SNCA*	Elevated levels of APP and Aβ, and α-synuclein Impairment in γ-secretase activity	Gene editing (e.g., CRISPR – in development for humans)

Aβ, amyloid beta; AD, Alzheimer’s Disease; AMPA, receptor, α-amino-3-hydroxy-5-methyl-4-isoxazolepropionic acid receptor; APOE, apolipoprotein E; APP, amyloid precursor protein; CRISPR, clustered regularly interspaced short palindromic repeats; DLB, dementia with Lewy Bodies; NMDA receptor, N-methyl-D-aspartate; PSEN, presenilin; SNCA, synuclein alpha; SV2A, synaptic vesicle glycoprotein 2A.

#### APP, PSEN1, and PSEN2

Early-onset familial AD, which constitutes less than 1% of cases, can be triggered by highly penetrant mutations in genes encoding amyloid precursor protein (*APP*) on chromosome 21, presenilin 1 (*PSEN1*) on chromosome 14, and presenilin 2 (*PSEN2*) on chromosome 1 (89–91). Among the roughly 35 distinct *APP* mutations associated with AD pathogenesis are gene locus duplications and point mutations in the coding region, leading to amino acid substitutions. Duplication of the entire gene or locus results in elevated APP and Aβ levels, favoring the formation of Aβ plaques (92, 93). *PSEN1* and *PSEN2* are not only involved in γ-secretase but also in cleaving other type I integral proteins like the Notch receptor (94). Likewise, mutations in *PSEN1* and *PSEN2* hinder γ-secretase activity, causing an imbalance in the Aβ_1–42_ to Aβ_1–40_ ratio due to Aβ_1–42_ overproduction or Aβ_1–40_ underproduction, or a combination thereof. The Aβ_1–42_ to Aβ_1–40_ ratio is significant because an increase in this ratio increases the aggregation and neurotoxicity of the Aβ protein while a decrease in the ratio can decrease deposition (95–98). *APP*, *PSEN1*, and *PSEN2* mutations contribute to neural excitability by activating the mentioned mechanisms via elevated Aβ levels and amyloid plaque formation. It is important to note that early-onset AD is not only related to *APP*, *PSEN1*, and *PSEN2*. Alterations in these three genes only account for 5–10% of early-onset AD with remaining genes and risk factors still to be discovered and studied (89, 99–101). Beyond these known genetic variants causing AD, individuals with Down syndrome possess an extra copy of chromosome 21, housing *APP*, and face an elevated risk of early-onset AD and seizures (102, 103). Estimates indicate that more than 50% of people with Down syndrome will develop Alzheimer’s with symptoms emerging in their 50s and 60s (104, 105).

#### SNCA

Mutations in the *SNCA* gene, which encodes α-synuclein, lead to parkinsonian disorders, notably including DLB (106–108). Among the numerous mutations, A30P, E46K, G51D, and duplications and triplications of the *SNCA* gene, of specific interest is the A53T point mutation (106, 109, 110). Recent investigations have uncovered that mice expressing human α-synuclein with the A53T mutation manifest a phenotype akin to the human condition (110). They exhibit deficits in long-term potentiation and learning and memory. Furthermore, these mice display a left shift in electroencephalography (EEG) spectral power, mirroring the EEG slowing observed in patients with DLB (110–112). The EEG slowing and shift in spectral power to more delta signifies network dysfunction, a loss of cholinergic neurons, and symptoms of DLB (66, 68). Similarly, Morris et al. (66) demonstrated that neuronal overexpression of wild-type α-synuclein in transgenic mice (Thy1-SYN line 61) also leads to EEG slowing. Both of these models experience seizures and present molecular alterations in the hippocampus that suggest abnormal network excitability, including a depletion of calbindin in the dentate gyrus. These collective findings suggest that higher levels or dysfunction of α-synuclein may contribute to the neuronal hyperactivity found in DLB.

### Degeneration of cholinergic pathways

Acetylcholine is an ester of acetic acid and choline that is released by cholinergic neurons (113, 114). Acetylcholine plays a crucial role as one of the neurotransmitters implicated in cognitive functions like memory and executive function. In both DLB and AD, deficiencies in cholinergic activity are observable (115, 116). These deficiencies manifest as reduced acetylcholine levels and irregularities in the expression of nicotinic and muscarinic receptors. Notably, the extent of cholinergic deficits tends to be more pronounced in DLB when compared to AD, even though DLB typically exhibits less brain volume loss (49, 117). The decline of cholinergic neurons projecting to the cortex contributes to a deceleration of cortical oscillations as seen on EEG, resulting in a shift of spectral power from higher frequency bands (alpha, beta, gamma) to lower ones (delta, theta) (118, 119). DLB patients experience a more significant loss of cholinergic neurons, displaying more pronounced EEG slowing (49). Additionally, DLB patients demonstrate greater clinical improvement with the usage of common acetylcholinesterase inhibitors such as donepezil, rivastigmine, and galantamine compared to AD patients (120, 121). It is unknown whether neurodegeneration of cholinergic neurons contributes to hyperexcitability. However, animal models suggest that early changes in cholinergic tone could contribute to epilepsy in preclinical stages of AD. Interictal spikes have been observed during the rapid eye movement stage of sleep in Tg2576 mice expressing human amyloid precursor protein (APP) at a very young age (5 weeks old), long before the deposition of Aβ (122). After administration of muscarinic cholinergic receptor antagonist, atropine, the investigators observed a reduction in interictal spikes, suggesting that there may be a phase of high cholinergic tone, contributing to epilepsy, prior to reductions in acetylcholine (122). In contrast, donepezil, a cholinesterase inhibitor had no significant effect on interictal spikes (122). Another study using the APPswe/PS1dE9 mouse model presenting with spike–wave discharges (SWDs), showed that donepezil does not have a significant effect on epileptic activity whereas atropine decreases SWDs and results in EEG slowing (123). This information suggests that before the degeneration of cholinergic neurons in AD and DLB, there could be a phase of increased cholinergic tone that contributes to an increase in neuronal activity and epilepsy.

### Glutamate

Glutamate, among the most extensively studied neurotransmitters within the central nervous system, is a non-essential amino acid synthesized within neurons and glial cells using glucose and α-ketoglutarate. It is ubiquitously distributed throughout the brain (124). Glutamate holds significance in cognitive functions like memory and learning, playing a pivotal role in neuronal excitability by expediting swift synaptic activity in neurons—a process regulated by astrocytes and other glial cells (125). The distribution of glutamate across distinct brain compartments is orchestrated by specific transporters and enzymes accountable for its metabolism. Surplus glutamate is eliminated by glial cells through excitatory amino acid transporters (EAAT1, EAAT2) (126). Notably, reduced expression levels of EAAT1 and EAAT2 have been observed in cases of epilepsy (127, 128), while mutations in the *SLC1A3* and *SLC1A2* genes that encodes EAAT1 and EAAT2, can result in episodic ataxia 6, characterized by symptoms of epilepsy, long lasting ataxia attacks and headaches, and epileptic encephalopathies, respectively (129, 130).

Inside astrocytes, glutamate undergoes a transformation into glutamine, subsequently being released and taken up by the neuronal presynaptic compartment. There, it is converted back into glutamate, which then accumulates within synaptic vesicles via vesicular glutamate transporters (vGLUT1/vGLUT2). Although astrocytes are commonly discussed collectively, they are an extremely diverse cell population. A recently described subpopulation of astrocytes specifically mediates the release of glutamate (131). Ultimately, this intricate process facilitates highly efficient neurotransmission within tri or tetrapartite synapses (132, 133).

An imbalance in the expression of vGLUT1 was observed in post-mortem human brain samples at the advanced stages of both AD and DLB (134). Similarly, Liraz et al. (135) discovered reduced levels of vGLUT in the hippocampal neurons of APOE4 mice. Previous research studies have pointed to a decline in the capacity and protein expression of glutamate transporters, as well as a specific loss of vGLUT in AD patients (136–138). A postmortem study showed increases in EAAT1 levels in a subset of pyramidal neurons exhibiting degeneration in the AD brain (139), whereas another postmortem study and *in vitro* assay showed impaired function of EAAT2 in the AD brain (140). Pharmacological administration of riluzole increases glutamate transporter expression, and in the P301L mouse model reverses glutamate related alterations and associated cognitive decline (141). Consequently, elevated levels of glutamate contribute to excitotoxicity and neuronal cell death (142). These findings collectively suggest that as the disease advances, the transporters responsible for glutamate reuptake become less effective, potentially leading to increased neuronal excitability.

Glutamate toxicity primarily arises from an excessive influx of Ca^2+^ (143, 144). Dubinsky (145) demonstrated that hippocampal neurons exposed to toxic levels of glutamate maintained elevated Ca^2+^ levels for around 1 h before returning to baseline levels. As calcium signaling governs a spectrum of cellular processes, the outcome of Ca^2+^ overload entails the activation of catabolic enzymes like calpain I (146), phospholipases, and the release of arachidonic acid (147). This cascade results in an escalation of reactive oxygen and nitrogen species and the eventual collapse of neuronal cells through cytoskeletal degradation and membrane deterioration. Clinically, this associates with the progressive decline in cognition and memory, as well as brain atrophy in AD patients (148, 149). This is further evident in epilepsy where the seizures ultimately cause excitotoxicity by starting the aforementioned cascade and leading to neuronal cell death and loss (150, 151).

Upon release from synaptic vesicles, glutamate initiates the activation of diverse ionotropic (AMPA, kainate, NMDA) and metabotropic (mGluR1 and mGluR5 in group I, mGluR2 and mGluR3 in group II, and mGluR4,6,8 in group III) glutamate receptors, primarily located in the postsynaptic region (152). The overstimulation of these receptors contributes to the generation of free radicals, possibly as a result of the continued calcium influx, inducing oxidative stress and subsequently disrupting mitochondrial functions (152, 153). This mitochondrial dysfunction plays a role in initiating and advancing epilepsy by triggering sequences of apoptosis (154).

Recent research highlights NMDA receptors (NMDARs) as contributors to neuronal hyperexcitability, suggesting that abnormal activation of these receptors, particularly through Ca^2+^ influx, is implicated in hyperexcitability (155, 156). NMDARs possess a significantly higher permeability for calcium ions compared to other ionotropic glutamate receptors (iGluRs), thus facilitating hyperactivity through calcium influx (155, 156). Memantine, an NMDAR antagonist, has been found to reduce Ca^2+^ influx and improve cognition and behavior in moderate-to-advanced AD (157). On the other hand, direct links between AMPA receptors and epilepsy in AD and DLB are more limited. Elevated levels of AMPA receptors have been observed in the brains of various epilepsy types, in humans and animal models (158, 159), and there is evidence of changes in receptor function through increased levels of AMPA and NMDA receptor subunits in human and mouse epileptic brains (160, 161).

Studies such as that by Teravski et al. (110) involving A53T α-synuclein-expressing neurons have indicated postsynaptic dysfunction, including reduced amplitude of miniature postsynaptic currents and a lower ratio of AMPA to NMDA receptor currents. Such changes coincide with the development of epileptic activity in this model. If the loss of AMPA receptors occurs in GABAergic inhibitory neurons, this could enhance the activity of neurons receiving their projections, potentially leading to neural network hyperactivity in DLB. Further exploration of the roles of NMDA and AMPA receptors in AD and DLB could yield valuable insights into potential treatments for epilepsy associated with these diseases.

### Overactivation of mTOR Pathway

mTOR, mechanistic target of rapamycin, is a highly conserved serine/threonine protein kinase that forms two distinct complexes, mTORC1 and mTORC2. External triggers including energy, oxygen, DNA damage, and amino acids activate the mTOR complexes, and they are implicated in a breadth of physiological functions including cell survival, growth, proliferation, metabolism, protein synthesis and signaling (162). In the brain, mTOR expression is widespread, affecting many neuronal and glial cell types playing a role in axonal development, synaptic plasticity, and neuronal excitability (163). Another crucial role function of mTOR signaling is autophagy, the process of degrading and recycling components of dysfunctional cells and proteins (164). Recently however, the role of autophagy has been expanded and shown to affect neuronal excitability (165). An ATG5 deficient mouse model shows impairment and decreases in protein kinase A (PKA) signaling from the lack of PKA subunit turnover (165). In addition to increased excitatory neurotransmission, alterations in synapses and disruption in AMPA receptor function, seizures also present as a common phenotype in these mice. This further highlights mTOR’s myriad functions and its contribution to hyperexcitability and warrants further investigation in the context of neurodegeneration.

Overactivation and dysregulation of mTOR can result in severe pathological changes. mTOR association with hyperexcitability and seizures can be attenuated pharmacologically (125, 166, 167). mTOR hyperactivation is observed in Tuberous Sclerosis Complex (TSC) which presents with epileptic seizures and autism-like traits (162). Loss of the TSC gene in mouse models results in seizures and epilepsy that can be attenuated with the mTOR inhibitor, rapamycin (168). mTOR overactivation can also be activated by seizures evidenced by an increase in phospho-S6 expression in a kainic acid seizure mouse model (169). Inhibition of mTOR and restoration of the excitatory imbalance causing seizures and epilepsy may provide additional benefit for AD and DLB where hyperexcitability may participate in a positive feedback loop (40).

Pertaining to AD and DLB, postmortem examinations revealed increased mTOR activation in AD, DLB, and Parkinson’s disease and associations with deficits in autophagy (170–173). Seizures have been shown to both activate mTOR and worsen AD pathology and cognitive deficits. Rapamycin administration can attenuate cognitive deficits in AD models through an increase in autophagy and/or decrease in hyperexcitability, further linking overactivation of mTOR activity and its contribution to AD (174, 175). Within the context of hyperexcitability, mTOR in DLB may be underappreciated and understudied. Since autophagy deficits have been implicated in DLB, hyperexcitability may be a mechanistic link with mTOR. A better understanding of these mechanisms and connection to hyperexcitability in AD and DLB may allow for more targeted therapeutics beyond rapamycin in lessening the overactivation of mTOR and burden of its wide-ranging effects.

### Proteinopathies

#### Alpha-synuclein

Alpha-synuclein is a protein composed of 140 amino acids. It was initially identified in association with synaptic vesicles within the presynaptic nerve terminal and has demonstrated interactions with membranes (176, 177). This protein modulates synaptic transmission, influences the density of synaptic vesicles, and contributes to neuronal plasticity (178, 179).

Beyond its synaptic functions, extracellular alpha-synuclein has a pivotal impact on neuroinflammation, neurotoxicity, and the propagation of pathological changes (180). It is transported into the extracellular space following active secretion or release from dying neurons. The exact mechanism behind the secretion of alpha-synuclein is unknown. However, research by Paillusson et al. (181) indicated that enteric neurons can release it via conventional endoplasmic reticulum/Golgi-dependent exocytosis, which is driven by neuronal activity.

Clinical and experimental studies demonstrate that α-synuclein expression participates in epilepsy (182–185). Tweedy et al. (186) demonstrated hippocampal network hyperexcitability in young transgenic mice expressing human mutant alpha-synuclein. Yang et al. (185) identified anomalous accumulations of this protein in hippocampal samples taken from individuals with mesial temporal lobe epilepsy (MTLE). These deposits were correlated with the loss of neuronal cells and reactive gliosis, indicating a potential link between the presence of the protein and the pathological changes seen in MTLE. Another clinical study in children with epilepsy showed that higher levels of serum α-synuclein correlated with disease severity (182). In the same way that α-synuclein levels are associated with seizures in epilepsy patients, it may also be associated with epileptic events in AD and DLB. A mechanism by which α-synuclein contributes to epilepsy could be activation of astrocytes and microglia, enhancing glial proinflammatory activity cytokines, nitric oxide, and reactive oxygen species (184, 187). More investigations are warranted to determine whether lowering α-synuclein levels or inhibiting its aggregation in the brain modulates epilepsy.

#### Tau protein

The microtubule-associated protein tau predominantly resides within axons, where it plays a vital role in assembling microtubules. Tau can also be located in various neuronal compartments, such as somatodendritic regions and nuclei, and it is even detectible within glial cells (188, 189). In cases of pathology, tau undergoes hyperphosphorylation within neurons, diminishing its affinity for tubulin. This leads to the aggregation of tau into neuropil filaments or NFTs, giving rise to tauopathies (190–192).

Brain aggregates of hyperphosphorylated tau have been noted in patients with epilepsy as well as various models of epilepsy (40, 68, 193). This indicates that the abnormal aggregation of phosphorylated tau might play a role in the pathogenesis of epilepsy. Additionally, the tau protein seems to contribute to the development of epilepsy in the context of AD and DLB. Referencing Hwang et al. (40), endogenous tau acts as an enabler of hyperexcitability and seizures and, within the context of epilepsy and AD, a complex balance may occur in an attempt to decrease hyperexcitability. Total tau is reduced after 2 months in a status epilepticus (SE) model of epilepsy (194). After 4 months, tau levels return to normal while phosphorylation at tau sites S202/T205 is reduced by about 50%. This may highlight how tau changes in response to hyperexcitability over time in an attempt to reach homeostasis.

Numerous investigations have demonstrated that genetically altering tau or diminishing tau levels can result in an increase or decrease in seizures and epileptic activity across different animal models. Ablating both tau and Fyn in a mouse model shows robust neuroprotection from pentylenetetrazol, including increased seizure latency, reduced seizure stage, and reduced gliosis (195). Roberson et al. (196) demonstrated that reducing normal tau prevents the occurrence of spontaneous epileptiform activity across multiple lines of transgenic mice expressing human APP. Conversely, transgenic mice that overexpressed wild-type human tau or tau with an A152T mutation exhibit epileptiform activity and heightened susceptibility to seizures (197). The A152T tau mutation induces more pronounced network hyperexcitability compared to wild-type tau (197). *In vitro* studies using the rTg4510 mouse model, which features mutant (P301L) human tau, revealed increased neuronal excitability in the cortex’s layer 3 even before the formation of NFTs. In the CA1 region of the hippocampus, pyramidal neurons display heightened firing, while inhibitory interneurons exhibit reduced activity, indicating a breakdown in inhibitory synaptic transmission (198, 199).

Recent investigations involving mice expressing human α-synuclein with the A53T mutation highlighted that endogenous tau contributes to hyperexcitability and that epileptic activity diminishes in the absence of tau (68). Delving deeper into the pathways influenced by tau, Decker et al. (200) demonstrated that hyperphosphorylated tau could stimulate presynaptic glutamate release, resulting in hyperexcitability. The toxicity of glutamate has been linked to tau-mediated neuronal cell death and behavioral deficits in drosophila (201).

The observation that physiological endogenous tau levels in adult mice impact seizure susceptibility suggests that similar relationships might exist in humans, potentially influencing the risk of developing seizures. This supports the notion that reducing tau could contribute to preventing seizures (202) and offers an opportunity for pharmacological intervention targeting tau.

#### Amyloid beta (Aβ)

Amyloid precursor protein (APP) is a transmembrane protein encompassing a sizable extracellular domain and a smaller intracellular segment. Amyloid-beta (Aβ) peptides stem from the proteolytic cleavage of APP, sequentially catalyzed by β-secretase and γ-secretase. In pathological conditions, Aβ peptides amass into dense fibrillary plaques. Aβ has been demonstrated to incite network dysregulation, culminating in heightened synchronicity and seizures. This increased neuronal activity, in turn, exacerbates neurodegeneration (203). Recent investigations indicate that Aβ possesses epileptogenic properties and can significantly influence the trajectory of cognitive decline (12, 204). Ovsepian and O’Leary (205) proposed that seizures might foster the deposition of Aβ plaques. This epileptogenic potential of Aβ was validated in the APP/PS1 model, where neurons exhibiting epileptic discharges were found to colocalize with Aβ plaques (206). Evidence also suggests that Aβ could have epileptogenic effects even during pre-plaque stages. Hyperactivity among hippocampal neurons during the initial phases of Aβ pathology, when Aβ fibrils remain soluble, has been observed in APP/PS1 mice (122, 207). APP/PS1 mice also present with an increase in soluble and insoluble Aβ_1-42_ and an increase in seizure susceptibility with corneal kindling (208). Aβ oligomers, being synaptotoxic, might trigger epileptic discharges prior to plaque deposition (209). Exposure to Aβ oligomers can also lead to spontaneous neuronal firing in hippocampal neurons (210).

Studies indicate that Aβ-triggered neuronal epileptic activity is tied to alterations in voltage-dependent channels that regulate the neuronal membrane potential. In a drosophila model expressing human Aβ42, Ping et al. (211) demonstrated that fewer Kv4 channels in neurons promote hyperexcitability, while Kv2 and Kv3 channels remained unaffected. Other research has shown that Aβ can perturb calcium homeostasis by either stimulating voltage-gated calcium channels or creating membrane pores, thereby augmenting calcium influx (212). Additionally, Aβ can influence glutamate release. Talantova et al. (213) illustrated that Aβ interacts with α7 nicotinic acetylcholine receptors, leading to the release of astrocytic glutamate, which subsequently activates extrasynaptic NMDA receptors on neurons. Similarly, Zott et al. (214) employed Aβ-amyloidosis models to reveal that hyperactivity is initiated by dampening glutamate reuptake. Soluble Aβ oligomers hinder the uptake of glutamate and intensify extrasynaptic NMDAR activation. Thus, Aβ can trigger a sequence of molecular events culminating in neural hyperexcitability.

### Glia and neuroinflammation

Glial cells are brain defense cells comprising microglia, astrocytes, and oligodendrocytes. When stimulated, microglia and reactive astrocytes release modulators to facilitate the recovery of the tissue from damage (215, 216). However, the continuous stimulation of the glial network causes a cascade of molecular events leading to neuroinflammation (215, 217). Investigators have previously proposed that neuroinflammation stimulates heightened neuronal activity and seizures, and the disruption of glial immunoinflammatory function is considered a factor that could predispose to or play a role in the emergence of seizures (218, 219). Therefore, inflammatory mediators and epileptic seizures form a vicious positive feedback loop, reinforcing each other (220). This vicious cycle can be found in diseases with neuroinflammatory conditions such as AD and DLB and to be responsible for epileptogenesis.

Elevated concentrations of pro-inflammatory cytokines, notably interleukin-1β (IL-1β), IL-6, and tumor necrosis factor-α (TNF-α), have been linked to epileptic seizures (221). The increased concentration of pro-inflammatory mediators can participate in hyperexcitability by increasing glutamate release via decreasing levels of the astrocytic glutamate transporter EAAT2 (excitatory amino acid transporter 2) (222, 223). Before and after seizures, there is an increase in the levels of pro-inflammatory cytokines and the expression of their receptors in both glial cells and neurons (221). In epileptic and AD patients, TNFα levels are elevated in the brain (224, 225), and TNFα increases the sensitivity of AMPA and NMDA glutamatergic receptors in the postsynaptic neuron, leading to excitotoxicity (223, 226). TNFα also induces endocytosis of neuronal ionotropic GABA_A_ receptors, so that neurotransmission becomes more excitatory, leading to epilepsy (227). Furthermore, Xiaoqin et al. (85) found that the intracerebroventricular injection of IL-1β in rats leads to a reduction in cortical and hippocampal GABA concentration, while simultaneously increasing glutamate release. This alteration in neurotransmitter balance enhances the brain’s vulnerability to seizures.

Another potential mechanism underlying seizure activation is the engagement of TLR4 receptors (228). TLR4 acts as the primary receptor for the proinflammatory mediator High Mobility Group Box 1 (HMGB1). Activation of TLR4 via HMGB1 sets off seizures by initiating a Ca^2+^ influx subsequent to the phosphorylation of the NR2B subunit of the NMDAR. In support of this, Maroso et al. (229) revealed an elevation in TLR4 expression in hippocampal samples from individuals with drug-resistant temporal lobe epilepsy compared to control subjects. Furthermore, inflammation triggers the release of reactive oxygen species and reactive nitrogen species, thereby heightening susceptibility to seizures and intensifying the inflammatory milieu in the brain (230). This inflammatory environment gives rise to mediators like pro-inflammatory cytokines, transforming growth factor-β, and prostaglandin E2 (231). These mediators stimulate astrocytes and impact glutamate release, culminating in hyperexcitability.

Triggering receptor expressed on myeloid cells 2 (TREM2) are primarily expressed by microglia and play a role in the immune response. TREM2 expression in the brain has been found to be increased in AD and thought to provide an adaptive response to AD pathology, while reduction in TREM2 and mutant variants increases susceptibility to hyperexcitability and epileptic activity (232, 233). In regards to DLB, results are mixed as to whether soluble TREM2 is increased, and more research is needed to determine TREM2’s influence on DLB-related hyperexcitability (234–236).

In addition to releasing inflammatory mediators, glial cells, particularly astrocytes, play a role in maintaining ion balance by clearing extracellular potassium (K+) during neuronal repolarization. Wang et al. (237) demonstrated that the onset of seizures is linked to elevated extracellular K+ levels due to astrocytic activity. Notably, the protein expression of the astrocytic potassium channel Kir4.1 is diminished in both a mouse model of AD and in the brains of AD patients (238).

Astrocytes often undergo reactive changes, termed reactive astrocytosis, characterized by increased astrocyte size and number. These changes are frequently observed alongside neuronal loss and synaptic reorganization (239). Reactive astrocytosis is present in conditions such as epilepsy, AD, and DLB, and it might contribute to neural hyperexcitability by influencing the function of astrocytic membrane K+ channels (240–242). In light of these insights, understanding the underlying mechanisms of inflammation in the development of epilepsy could pave the way for the discovery of promising antiseizure medications.

### GABAergic dysfunction

Gamma-aminobutyric acid (GABA) serves as the primary inhibitory neurotransmitter within the central nervous system. It is synthesized by the enzyme glutamic acid decarboxylase (240). This neurotransmitter is primarily found in interneurons that establish synapses on cell bodies and nearby axon segments. Released into the synaptic cleft, GABA exerts its influence through activation of GABA_A_ and GABA_B_ receptors. GABA_A_ receptors function as ligand-gated ion channels, promptly inducing inhibition by enhancing chloride influx into cells. In the context of AD, studies have shown moderate reductions in GABA_A_ receptors within the brain (243, 244). GABA_B_ receptors, on the other hand, are G protein-coupled ion channels that augment extracellular potassium transport while concurrently decreasing calcium influx. Mutations in genes encoding GABA receptor subunits have been linked to a range of epileptic disorders (245).

Investigations point to a potential mechanism involving GABAergic dysfunction contributing to hyperexcitability by influencing voltage-gated sodium channels. Studies by Verret et al. (246) and Hamm et al. (247) demonstrated variable decreases in Nav1.1 and Nav1.6 within hippocampus and somatosensory cortex mouse models of AD. These channels enhance gamma oscillations during exploration, which can help suppress epileptiform discharges. Consequently, Nav1.1 and Nav1.6 hold potential as targets for addressing epileptic seizures in the context of AD.

## Conclusion

In conclusion, hyperexcitability in AD and DLB arises from a combination of multiple factors working together to disrupt regulation of neuronal excitability. In this context, we observe that genetic predispositions, initial elevations in cholinergic activity, excessive calcium influx causing glutamate toxicity, heightened NMDA and AMPA receptor sensitivity, overactivation of mTOR, disruptions in calcium homeostasis due to Aβ, tau, and α-synuclein, hyperstimulation of microglia and astrocytes, and GABA dysfunction collectively contribute to the promotion of hyperexcitability in AD and DLB. By understanding the specific dysfunctions within these pathways, it becomes possible to develop targeted therapeutic strategies aimed at restoring proper neuronal excitability. Such interventions hold the potential to alleviate the symptoms associated with these neurodegenerative disorders, offering hope for improved treatments and better quality of life for affected individuals.

## Author contributions

MV: Writing – review & editing. KA-O: Writing – review & editing. KV: Writing – review & editing.
